# Statistical Mutation Calling from Sequenced Overlapping DNA Pools in TILLING Experiments

**DOI:** 10.1186/1471-2105-12-287

**Published:** 2011-07-14

**Authors:** Victor Missirian, Luca Comai, Vladimir Filkov

**Affiliations:** 1 Department of Computer Science, UC Davis, 1 Shields Ave., Davis, CA 95616, USA; 2 Department of Plant Biology and Genome Center, 1 Shields Ave., Davis, CA 95616, USA

## Abstract

**Background:**

TILLING (Targeting induced local lesions IN genomes) is an efficient reverse genetics approach for detecting induced mutations in pools of individuals. Combined with the high-throughput of next-generation sequencing technologies, and the resolving power of overlapping pool design, TILLING provides an efficient and economical platform for functional genomics across thousands of organisms.

**Results:**

We propose a probabilistic method for calling TILLING-induced mutations, and their carriers, from high throughput sequencing data of overlapping population pools, where each individual occurs in two pools. We assign a probability score to each sequence position by applying Bayes' Theorem to a simplified binomial model of sequencing error and expected mutations, taking into account the coverage level. We test the performance of our method on variable quality, high-throughput sequences from wheat and rice mutagenized populations.

**Conclusions:**

We show that our method effectively discovers mutations in large populations with sensitivity of 92.5% and specificity of 99.8%. It also outperforms existing SNP detection methods in detecting real mutations, especially at higher levels of coverage variability across sequenced pools, and in lower quality short reads sequence data. The implementation of our method is available from: http://www.cs.ucdavis.edu/filkov/CAMBa/.

## Background

TILLING (Targeting Induced Local Lesions IN Genomes) [1] is a reverse genetics approach to detect effects of globally induced mutations in a population and identify the individuals that have mutations in genes of interest. As long as the DNA sequence of the target gene is known and the organism of interest can be mutagenized, TILLING provides mutations in species where tools applicable to other model systems are unavailable. Importantly, organisms amenable to TILLING include both commercially valuable species such as rice [2], wheat [3,4], soybean [5,6], brassica [7], oat [8], and melon [9], and species important for research such as medaka [10], zebra fish [11], fruit flies [12], arabidopsis [13] and nematodes [14].

Furthermore, TILLING produces allelic series of decreasing function allowing functional characterization of genes whose knock-outs are lethal [15,16].

**Figure 1:**
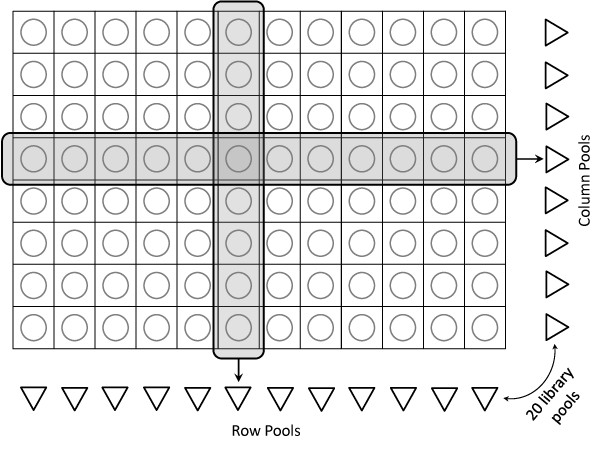
**Bi-dimensional arrangement of the overlapping pools experiments**. There are 96 wells and 20 pools (12 column- and 8 row-pools) in our bidimensional pooling scheme. Thus, each individual is present in two pools.

The TILLING approach is based on the ability to detect rare mutations in large populations and thus requires the use of methods that can detect a mutant allele in a pool where it is diluted by many wild-type alleles [17]. Previously, TILLING identified mutations in 8-fold pools of mutagenized individuals by detecting mismatches between annealed wild-type and mutant DNA strands. Subsequent to identification of a positive pool, all members of the pool were tested to identify the mutant individual. TILLING -by -Sequencing [18] leverages the Illumina sequencing platform and an overlapping pooled experimental design. It follows up the mutagenesis with deep sequencing of pools of individuals or populations of interest. Because of the high throughput of current sequencing technologies, deep sequencing to hundred and thousand fold coverage is possible [19]. This, in theory, should allow identification of rare mutations present at very low frequency in a sample, such as when a single heterozygous individual is present in a pool of 96 (1 mutant/192 alleles). Discovery, however, requires resolving true signal from sequencing noise. To identify both the mutations and individuals that carry them at high resolution, overlapping pools of individuals are used. TILLING-by-Sequencing uses a practical and effective overlapping pools design, where pools of individuals overlap in their DNA content in such a way that each individual's DNA is present in exactly 2 pools. We call this setup *bi-dimensional pooling*, and illustrate it in Figure 1. This design is readily extensible to multi-dimensional pooling, where each individual's DNA is present in 3 or more pools [18]. The general computational problem which arises from TILLING-by-Sequencing is: given a stretch of DNA from a reference genome and a set of deep sequenced, bi-dimensionally overlapping pools, identify the positions with mutations along the DNA and their individual carriers, or, equivalently, the position and the row- and column-pool for each mutation. Any solution to this problem would focus on identifying significant differences between the reference genome and the sequenced DNA. The problem is complicated by sequencing noise (false calls that cannot be recognized by simple sequencing quality criteria), the inter-dependency of pools of the experimental setup, the infrequency of mutations' occurrences with respect to the size of the population under study, and also by variability, or non-uniformity in the sequencing coverage, which is not uncommon for 2nd generation sequencing technologies [20].

**Figure 2:**
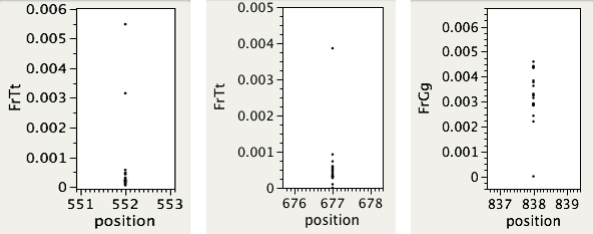
**Example base positions with mutations in the data of varying difficulty for identification**. Three mutations ordered, left to right, by increasing difficulty to identify visually. Left and middle, *C *→ *T *mutations at positions 552 and 677, respectively, in wheat genes APHYC and AVRN, resp. Right, an *A *→ *G *mutation at position 838 in rice gene OsRDR2.Each dot in the plots is a library pool, and on the y-axis is the frequency of the base to which the reference has been mutated.

For example, in a bi-dimensionally pooled experimental design, a mutation in a single individual is expected to cause a higher base change frequency in one row and one column pool, and many mutations can be recognized in this way, by visual detection of outliers. In Figure 2 we show the base change frequency for each pool at three positions with confirmed mutations from mutagenized wheat and rice. From left to right, there is apparent increased difficulty in identifying a mutation. The accuracy of calls made by visual inspection depends on the sequencing coverage, or number of nucleotide calls per position per pool. Given a fixed probability of base change due to error (with respect to a reference genome), at high coverage levels pools with real mutations will usually stand out clearly from the noise. As coverage drops however, in the absence of a real mutation a larger range of base change frequencies may reasonably occur by chance thus increasing the number of false positives. The visual approach cannot distinguish these cases because it does not take coverage levels into account, so a single gene that has low coverage on a few libraries can cause a high overall false positive rate.

Here we propose a new method, *Coverage Aware Mutation calling using Bayesian analysis*, *CAMBa*, (read like the dance) which directly considers the pooled setup and sequencing coverage levels when calculating mutation and noise probabilities. Using data from two TILLING experiments, one with lower sequencing coverage variablility and data quality and the other with higher, we validate CAMBa's efficacy in identifying mutations, and demonstrate that it does at least as well as other mutation calling methods, and that it outperforms significantly the other methods on sequence data of lower quality and higher variance in coverage across pools.

### Related Work

Several methods exist for identifying mutations in pooled experiments. Rigola et al. [21] use a Poisson distribution based outlier approach to identify mutations and natural variations in individuals using bi-and three-dimensional pooling schemes coupled with high-throughput sequencing. Shental et al. [22] apply Compressed Sensing to create a variant detection technique, ComSeq, which can handle computationally optimal pooling schemes. Unlike the Poisson outlier method, CAMBa considers the experiment setup and models configurations that could have yielded the observed data. CAMBa uses sequencing coverage and bi-dimensional pool overlap as model features, whereas ComSeq uses only base change information, and a more general pooling approach. We compare the performance of these two methods to that of CAMBa in the Results section.

A variety of approaches exist for calling SNPs from non-overlapping pooled samples, e.g. VarScan [23], CRISP [24], SNPseeker [25], the MAQ alignment tool [26], and others; and non-pooled samples, e.g. POLYBAYES [27], PolyScan [28], the method by Stephens et al. [29], and others. Our approach, CAMBa, is specifically geared to working on pooled experiments with overlap between the DNA pools, i.e. DNA from the same individual is present in two pools. That is not the case for these other approaches, so we could not compare them directly to CAMBa. Moreover, these other approaches identify mutations but not the individuals in the populations that carry them. We modified VarScan and CRISP, the best performing ones from a previous study [24], in order to compare them to CAMBa and report those studies in the Results section.

Overlapping pool designs for high-throughput resequencing have been recently proposed by Prabhu and Pe'er [30], where they focus on optimizing overlaps to increase design efficiency (as compared to optimal), lower necessary sequencing coverage, decrease false positives and false negatives, and identify mutation carriers with lower ambiguity. They do not provide software for testing their designs and it is not immediately clear that their designs could easily fit into standard wet lab protocols (e.g. with respect to standard well plates, etc.). Our overlapping pooling scheme can be evaluated in their theoretical framework, and in terms of the "code efficiency" it is 50% worse than the theoretically optimal binary design (although it is not clear if that optimum is achievable in practice).

## Methods

In a bi-dimensional pooling setup we have *n_w _*wells, *i_well _*individuals pooled per well, *i_l _*individuals in library *l*, reference base *r *at the current position, the probabilities *p_c _*and *p_nc _*with which a mutagen will induce a specific canonical (*G *→ *A *or *C *→ *T*) or non-canonical base change at a given position in a single individual, and the fraction of induced mutations *t_z _*for each zygosity *z*.

The input data, *D*, is comprised of a set, *L*, of row and column libraries of short reads, corresponding to the pools of sequences of interest. The reads for each library are aligned to their reference sequences (using, e.g., MAQ (Mapping and Assembly with Quality) alignment tool [26]), associating each position in each sequence with a set of nucleotide calls: either the reference nucleotide base, *r *or a base change *r *→ *m*, *m ≠ r*. In the alignment, or pileup, of reads, for each position we count the total number of reads, i.e. coverage, in a given library, denoted by *n_l_*, and separately the number of reads that have base *b *at that position in that library, *k_lb _*(so *n_l _*= *k_lA _*+ *k_lT _*+ *k_lC _*+ *k_lG_*).

To find the carriers and the mutations, given the data, *D*, and the experimental setup, for each sequence position we model the posterior probabilities of each possible mutation in each well. We assume that at most one individual will have a mutation at any given sequence position. ^1 ^Thus, at most one well can have a mutation, and that mutation will be for one specific base change. We denote these possibilities, or *configurations*, as *c_w, m_*, where *w *is the well, and *m *is the base change from reference (since there are three possibilities for *m*, the number of possible *c*'s is 3 times the number of column libraries times the number of row libraries in the design). The probabilities corresponding to the configurations are *p*(*c_w, m_*|*D*). We call a mutation at a given position if the probability of at least one mutant configuration *c_w, m _*exceeds a predefined threshold indicating that well *w *contains an individual with base change *m *at the current position. If more than one *c_w, m _*pass the threshold, then the one with highest probability is chosen. The threshold is determined based on the expected number of mutations in an experiment, as described in the Methods section.

In the following we calculate the probabilities *p*(*c_w, m_*|*D*). Since the experimental procedure makes the expected number of heterozygous mutations equal to twice the number of homozygous mutations, we further distinguish configurations by zygosity, and use *c_w, m, z _*to model heterozygous, *z *= *het*, and homozygous, *z *= *hom*, mutations separately, and *p*(*c_w, m_*|*D*) = *p*(*c_w, m, het_*|*D*) + *p*(*c_w, m, hom_*|*D*).

We compute the posterior probability *p*(*c_w, m, z_*|*D*) of a given configuration, using Bayes' Theorem:

where *C *is the set of all possible configurations *c_w, m, z _*at the given position. Since we exclude all configurations with more than one mutant individual for the current position, the sum of the prior probabilities *p*(*c'*) do not add up to 1, but normalizing does not affect the result. Thus all we need to calculate are the terms in the numerator.

To compute *p*(*D|c_w, m, z_*) we need to accurately determine the position-specific sequencing error rate for different base changes. Given configuration *c*, we can estimate the rate at which the reference base *r *is read as *m*, *r*_*r*→*m*_, for a given position, as

where *L_c _*is the set of libraries that do not contain a mutation *m *at the given position, for the given *c*.

Now, to calculate the base change frequencies expected from a real mutation, we compute *m_lc_*, the fraction of mutant alleles in library *l *under configuration *c*, from the number of individuals *i_l _*in library *l *and the zygosity *z *of the candidate mutation.

Given the sequencing error rates and mutation allele frequencies, we can now compute *r_lmc_*, the expected rate of reading a given base change *m *at the current position in a given library *l*, for configuration *c_w, m, z_*: *r_lmc _*= (1 *m_lc_*)*r*_*r*→*m, c *_+ *m*_*lc*_.

To this, we apply the Binomial distribution to estimate the conditional probability of observing *k_lm _*reads having base *m *at a given position, given *n_l _*coverage for that library and configuration *c_w, m, z_*.

Finally, assuming that all base change counts are independent, we have

To compute *p*(*c_w, m, z_*) we start from our assumption that at most one well can contain a mutation at any given position. The prior probability of the presence of a particular base substitution *r *→ *m *at the current position in exactly *i *out of the *i_well _*individuals in a given well can be estimated as:

where *B*(*i|i_well_*, *p_c_*) is the Binomial distribution, i.e. the probability of having *i *successes out of *i_well _*trials given an individual success probability of *p_c_*.

A given well has prior probability *p_tm _*= *p*_1*m *_of being a mutant well for base change *m *and *p_fm _*= *p*_0*m *_of being a non-mutant well for base change *m*. The prior probability of a given configuration *cw, m, z *is the product of the prior probabilities of each well *w *with respect to each possible base change for the given position, multiplied by the fraction of induced mutations with the given zygosity, if applicable. Converting this description to a formula gives us , where *m*, *m'*, and *m" *are the three possible base changes at the given position. Note that these probabilities are the same for all wells (i.e. they don't depend on *w*).

### Pre-processing

For each library, we compute a low-quality cutoff for base calls to be one standard deviation below the mean quality of the reference base calls. We do not search for candidate mutations at a position if the expected value of the total coverage there over all but two libraries is less than 10,000 (corresponding to a min coverage per individual of 7.23), to avoid inaccurate estimates of *r*_*r*→*m*_.

The orientation bias of a specific base is the ratio of reads mapping onto the forward strand to those mapping onto the reverse strand of the sequence. If the reference base orientation bias for a given library at the current position is different from the orientation bias of base change *m *with pvalue *<*0.01, then we set *p*(*D|c_w, m, z_*) = 0 to exclude each configuration *c *for which a well represented in that library is a mutant well for base change *m*. We also set *p*(*D|c_w, m, z_*) = 0 for these configurations if the reference base orientation bias for the given library is greater than 10 or less than 0.1, since a strong reference base orientation bias can make it difficult to detect a significant difference between the orientation biases of the reference base and a given base change. In addition, if a given library has more base reads for the candidate base change than for the reference base at the current position, then we set *p*(*D|c_w, m, z_*) = 0 for each configuration where a well in that library is a mutant well for any base change.

### Number of Predictions

We construct our initial estimate of the number of real mutations in a given experiment by adding up the probabilities of each possible induced base change at each position across all TILLING sequences in all individuals, where the probability of a given canonical or non-canonical base change is determined from CEL I screening of an experiment on the same organism using the same mutagen [2,4], as described in the data section below. By this method, we estimate 47 real mutations in Rice and 69 real mutations in wheat. Since CEL I has a significant false negative rate, we correct our initial estimate using additional validation information from the wheat experiment. When an older version of our approach was run on the wheat experiment, 8 of the 10 predictions ranked 86 to 95 were tested and all 8 were confirmed. We drop below this ranking to give the semi-conservative estimate of 107 real mutations. We divide 107 by 69 to get a candidate scaling factor of 1.55. We predict the number of real mutations for a given experiment to be 1.55 times our initial estimate of the number of real mutations from CEL I screening. The predicted number of mutations is 107, by definition, for wheat, and it is 75 for rice. This determines our threshold.

The above approach for determining the appropriate threshold yields very good bounds for our data and can be applied whenever previous CEL I screening experiments have been done. In the absence of such prior experiments, one can apply the following method, although the results may include higher false positive rates. The false positive rate at a given number of predictions can be estimated by running CAMBa using as input a scaled down bi-dimensional arrangement using only the row pools. E.g., the row pools in the new scheme could be half of the actual row pools, and the new column pools could be the other half of the original row pools. Since we expect few or no instances where the same mutation occurs in two independent row libraries, the number of row/row calls serves as an upper bound on the number of false positives among the row/column candidates. Similarly, we could scale down the original arrangement using the original column pools instead of the row pools. We scale up by the ratio of the number of row/column pools versus the number of row/row pools, and choose the largest number of candidates for which the estimated false positive rate is nearest to our goal threshold. As an illustration of this method, we split the wheat data set 12 column pools into two groups of 6 pools each, and ran CAMBa on this new bi-dimensional pooled data of 6 rows and 6 columns. At a false positive rate of 0.05, this method yields a threshold for CAMBa of 105 mutations.

We note that although CAMBa yields posterior probabilities for each of thousands or tens of thousands of positions, we never use hypothesis testing to determine the threshold in either of the two approaches above, and thus we need not correct for multiple hypothesis testing.

Due to the apparent bimodality of the calculated posterior probabilities, and their clustering around the values of 0 and 1, we apply the following function to transform *t*, the posterior probabilities returned by CAMBa:

*F*(*t*) is effectively the log posterior probability. For both the rice and wheat TILLING-by-sequencing experiment, the predictions of CAMBa and the other methods are compared against the corresponding set of confirmed mutations.

## Results and Discussion

Using data from two TILLING-by-sequencing experiments we analyze the performance of *CAMBa *and compare it to those of other approaches. We investigate the effect of sequencing quality, sequencing coverage variability, and the overlapping pool design on the fidelity of our and the other methods in resolving mutations from the data.

### Two TILLING Experiments

The TILLING-by-sequencing setup in one of our labs (Comai) uses the mutagen ethyl methanesulphonate (EMS) or the combination of sodium azide and methyl-nitrosourea (Az-MNU) to induce mutations in a population of 1500-6000 individuals. M2, the selfed product of mutagen-treated individuals, are inventoried as DNA. The mutations in this material will be heterozygous in 2/3 of the cases and homozygous in the rest. Units of 768 individuals arrayed in a 96 well-plate, 8 individuals per well, are then screened. The row and column samples are pooled to yield 8 row- and 12 column-pools (for a total of 20 pools), as in Figure 1.

DNA from each of the 20 pools is PCR-amplified with primers designed to amplify 1-1.5 kb DNA segments from up to 40 genes of interest, and subsequently sequenced using Illumina Genetic Analyzer apparati. The reads are then mapped onto reference genomes.

Using this setup a total of 13 rice genes (avg. TILLING seq. length = 1393 bp) and 5 wheat genes (avg. TILLING seq. length = 934 bp) of interest were sequenced using Illumina GA machines to look for mutations in a population of 768 individuals. The reads on the average were of length 35 bp for the rice and 40 bp for the wheat data. There was a significant difference in the read quality between the two: the rice sequence had an average Phred quality score of 13 and the wheat of 31. There was also a larger variance in coverage between individual libraries in the rice data set than in the wheat data set. Also, on average, the coverage was 140 × per individual in rice and 270 × in wheat. The differences in quality, coverage, etc. between these two data sets make them very good case studies for our method. The TILLING-by-Sequencing experimental methodology and these two data sets are described in full elsewhere [18] (where they are called respectively Experiment 1 and Experiment 2).

Using prior TILLING experiments we determine the probabilities of a canonical mutation, *p_c_*, non-canonical mutation, *p_nc_*. We assume position independent values for *p_c _*and *p_nc_*, as indicated by prior experiments [2-4]. We estimate *p_c _*and *p_nc _*from *i_c _*and *i_nc_*, the induced mutation rates for canonical and non-canonical mutations, and *p_til_*(*b*), the fraction of TILLING reference sequences with base *b*, for each *b *(in prior corresponding experiments): *p*_*c *_= *i*_*c*_/*p*_*tilG*, *C *_and *p*_*nc *_= *i*_*nc*_/*p*_*nc *_(2*p*_*tilG, C *_+ 3*p*_*tilA, T*_). We compute *i_c _*and *i_nc _*using previously described methods [2,4]. Thus, we get for rice, *p_c _*= 5.6 × 10^-6 ^and *p_nc _*= 4.93 × 10 ^-7^, and for wheat, *p_c _*= 3.88 × 10^-6 ^and *p_nc _*= 0. The fraction of heterozygous mutations is *t_het _*= 2/3, and homozygous mutations is *t_hom _*= 1/3.

To evaluate the performance of the mutation calling algorithms, we used two sets of mutations, one set for wheat and one for rice, which have been previously confirmed using an independent method (PCR amplification followed by Sanger sequencing) [31]. In total we had 39 confirmed mutations from the wheat experiment and 11 from the rice experiment. ^2 ^We note that the confirmed mutations are a fraction of the total expected mutations in these data sets. Ideally, all predicted mutations should be tested, but practical resource constraints dictate limits on the validation.

### Validation of CAMBa's Performance

We ran CAMBa on each of the two data sets, TILLING on rice and TILLING on wheat, for a number of different mutation prediction thresholds, including the recommended one (above) and a few others above it and below it. For each threshold, we noted the number of predicted mutations (Pred), the corresponding value for *F*(*t*), the number of predicted mutations that overlap with the confirmed ones (Conf) (11 in rice, 39 in wheat), and the number of false positives (FP), false negatives (FN), and the sensitivity (Sens) and specificity (Spec) at that threshold. The results are given in Table 1.The last four columns were calculated by estimating the false positive and false negative rates from the overlap and the expected mutations, 107 in wheat and 75 in rice, and the total sequenced DNA positions, 18109 bp in rice, 4670 bp in wheat. We assume that the confirmed mutations have been randomly chosen from the set of all mutations, thus, we scale the true positives by 107/39, with the restriction that the number of False Positives must be at least 0. At the recommended thresholds, in wheat this gives *TP *= 99, *FP *= 8, and *FN *= 8, for a false negative rate of 7.5 × 10^-2 ^i.e. sensitivity of 92.5%, and a false positive rate of 2 × 10^-3 ^i.e. specificity of 99.8%. Similarly, in rice, CAMBa has sensitivity of 90.7% and specificity of 99.96%. The good performance of CAMBa is evident around the recommended thresholds.

**Table 1:** Performance of CAMBa at various thresholds on the Rice and Wheat data sets

Rice							Wheat						
*F*(*t*)	Pred	Conf	FP	FN	Sens	Spec	*F*(*t*)	Pred	Conf	FP	FN	Sens	Spec
0	308	11	233	0	100.00	98.71	-10	310	37	208	5	95.33	95.44
1	131	11	56	0	100.00	99.69	-5	172	36	73	8	92.52	98.40
2	75	10	7	7	90.67	99.96	0	107	36	8	8	92.52	99.82

3	54	10	0	21	72.00	100.00	5	92	33	1	16	85.05	99.98
4	46	9	0	29	61.33	100.00	10	81	31	0	26	75.70	100.00
5	40	7	0	35	53.33	100.00	15	59	21	1	49	54.21	99.98

Performance of CAMBa at various thresholds on the Rice (left) and Wheat data sets. *F*(*t*) = CAMBa threshold, Pred = # predicted mutations at that threshold, Conf = # of the predicted mutations which overlap with the confirmed mutations (11 in rice and 39 in wheat), FP = # false positives, FN = # false negatives, Sens = Sensitivity, Spec = Specificity. We underscore the line associated with the recommended number of predictions for both Rice and Wheat. We restrict our estimate of the number of False Positives to be at least 0.

It is notable that in rice, CAMBa predicted correctly 10 out of the 11 confirmed mutations at those thresholds. This is strong evidence that given lower quality data CAMBa can utilize the overlapping pools experimental setup to its advantage better than the other methods could. Even the much lower read data quality of the rice data (as given by the Phred quality scores above) does not seem to affect CAMBa's performance.

At the recommended threshold of 107 mutations in wheat, CAMBa predicted correctly 36 out of the 39 confirmed mutations. Looking closer in the sequence data for the 3 false negatives, we found out that one of them is due to strand-specific bias which resulted in wrong frequencies of base changes, and another was due to under-sequencing; the third showed up in the table at a much higher threshold.

### Methods for Comparison

We compare the performance of CAMBa to those of a number of methods, which we describe next. Not all of the other methods predict both the mutated positions and the individual carriers. Those that do not were either run separately on individual libraries or only predict the mutation positions. Hence, either they or CAMBa have been modified to allow for the comparison. In each case we specify the modification undertaken.

We devised the *Outlier method *as a naive competitor to CAMBa. It is inspired by simple visual identification of a row and column library pair that stand out from the rest in terms of the frequency of a given base change, and uses the same preprocessing techniques as CAMBa. When considering a given position in a TILLING sequence, if at least one well has a score greater than a fixed threshold *t *on some base change, then we predict a mutation for the base change and well combination with the highest score. For a given well *w *and base change *m*, we find the z-score of the *r *→ *m *base change frequency for both the associated row and column library with respect to the distribution of the *r *→ *m *base change frequencies for the remaining libraries, and we set the score of well *w *on base change *m *to be the lower of these two z-scores. We add 0.0001 to the sample standard deviation to avoid division by zero.

The *Poisson outlier method *is described in a TILLING-by-sequencing pipeline by Rigola et al. [21]. This method consider only G to A and C to T base changes for the purposes of mutation detection. Since MNU can induce any type of base change in rice, we modified the Poisson outlier method to search for all possible base changes when detecting mutations in rice. Rather than following the procedure for detecting natural variation, which considers all base changes as a whole, we tested for each base change individually, to reflect the assumption that an individual will have at most one mutation at a given position.

*VarScan *is a SNP identification method in individual or pools of massively parallel sequence data by Koboldt et al [23]. It identifies variants based on read counts, base quality, and allele frequency. VarScan does not take into account overlap in pools and it does not identify the individuals carrying the mutations. To compare it to CAMBa in a bi-dimensional setting, we ran VarScan separately on each row pool and column pool. We then took the intersection of the row and column calls.

*CRISP *[24] is a statistical method for variant detection in pooled DNA samples, shown to dominate a number of other methods in a direct comparison of SNP detection ability [24]. While similar to CAMBa at the modeling level, we note that CAMBa was developed independently and was used in our labs for more than 6 months before CRISP was published. Additionally, pool overlap is inherent to CAMBa's model, while CRISP does not utilize overlapping pools. Also, in contrast to the other competing methods that we consider, CRISP does not identify the mutation carriers. Thus, in the comparisons, we considered only the position and base change of each candidate returned by CRISP, while requiring the other methods to also predict the correct carrier. This decision allows a more fair comparison between CAMBa and the other methods, though giving CRISP a slight advantage. As with the others, we only include results for CRISP on its default parameters.

*ComSeq *[22] is a computational technique that identifies infrequent variants in complex pools. In contrast to CAMBa and the Poisson method [21], ComSeq allows for the use of computationally optimal pooling schemes [22]. Given the particular individuals contained within each pool and the observed frequency of a particular base change in each pool, ComSeq applies the theory of Compressed Sensing to infer which individuals have that base change. ComSeq does not appear to be designed to handle experiments with multiple individuals per well, so to simulate the presence of one individual per well, we multiplied the base change frequencies in each library by the number of individuals per well, resulting in increased sensitivity on both the Rice and Wheat data sets. ComSeq has no user parameters to vary and does not rank its predictions.

We also attempted comparisons with MAQ [26] but we could not get any mutation predictions on our data sets with their default settings.

### Comparison to Other Methods

We ran CAMBa versus the mentioned competing methods on their default settings, using our set of confirmed mutations and our prediction of the total number of real mutations to estimate the specificity and sensitivity of each method.

To ensure a fair comparison with CAMBa, we post-process all competing methods to return at most one mutation per position, selecting the best scoring candidate mutation for that position, where applicable. VarScan (when run without parameters) and ComSeq can return multiple candidates at a given position that cannot be distinguished by score, so in order to give them the best possible advantage, when one of their multiple predictions is a confirmed mutation, we allow them to always predict that confirmed mutation.

In the Wheat data set, we adjusted the competing methods to only consider the possibility of EMS canonical mutations, reflecting known information that is used in CAMBa. This filtering is accomplished by never considering EMS non-canonical base changes in the Poisson and Outlier methods, which we implemented by hand, and by post-processing the results of the other competing methods.

In Table 2 we show the number of predictions of each method when run on its default parameters, the overlap between those predictions and the confirmed set of mutation candidates, the false positives, false negatives, sensitivity, and specificity. The last four columns were calculated by estimating the false positive and false negative rates from the overlap and the expected mutations, as illustrated above.

**Table 2:** Performance comparison of CAMBa to other methods using default parameters on Rice and Wheat

	Rice						Wheat					
Method	Pred	Conf	FP	FN	Sens	Spec	Pred	Conf	FP	FN	Sens	Spec
CAMBa	75	10	7	7	90.67	99.96	107	36	8	8	92.52	99.82
Outlier	75	8	20	20	73.33	99.89	107	36	8	8	92.52	99.82
CRISP	0	0	0	75	0.00	100.00	121	32	33	19	82.24	99.28
VarScan	3	0	3	75	0.00	99.98	30	10	3	80	25.23	99.93
ComSeq	9599	10	9531	7	90.67	47.15	154	36	55	8	92.52	98.79
Poisson	6889	8	6834	20	73.33	62.10	218	35	122	11	89.72	97.33

Performance comparison of CAMBa to other methods using default parameters, on the Rice (left) and the Wheat data sets. The column abbreviations are as in Table 1. All methods are evaluated on their ability to predict the carrier of each candidate mutation, except CRISP, which cannot make such predictions.

On the rice data set, CAMBa dominates the other methods clearly. CRISP and VarScan return very few or no predictions on the Rice data set, most likely due to the lower sequencing quality. ComSeq and the Poisson method both return a large number of false positives.

On the wheat data set, we see greater overlap between the predictions of CAMBa and the other methods. This is consistent with the fact that the wheat data set is of higher quality, and thus mutations are easier 13 to identify.

### Coverage Variability over Libraries (Pools)

CAMBa shows a small performance advantage over the other methods on the wheat experiment, which has consistently high coverage levels across all libraries and between genes. In contrast, CAMBa has a very clear advantage in the rice experiment, and here we investigate the difference. While we found an explanation that accounts for our observations of the Outlier method, the other competing methods do not necessarily conform to the same rule.

**Figure 3:**
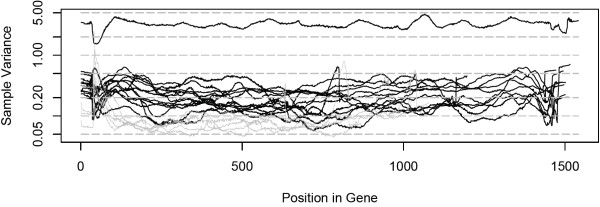
**Variance in coverage across libraries in the data**. Normalized variance of coverage levels across libraries in TILLING genes in rice (black) and wheat (gray). HLP1 is on top.

In Figure 3 we show the coverage variance across libraries for all genes in both experiments, wheat in gray, and rice in black. It is apparent that the black lines are overall higher on the plot than the gray lines, especially the line for gene HLP1. To test the hypothesis that insensitivity to sequencing coverage variability gives CAMBa an advantage over the other methods, we performed two computational studies. In the first one, we modify the rice data set to exclude the TILLING sequences for gene HLP1 which has both the lowest mean coverage and the highest coverage variability across libraries in this experiment. On this modified data set, the Outlier method performs as well as CAMBa, although the other competing methods do not significantly improve their performance, as shown in Table 3.

**Table 3:** Performance comparison of CAMBa to other methods using default parameters on Wheat with increased variance

Method	Wheat with increased variance					
	Pred	Conf	FP	FN	Sens	Spec
CAMBa	113	28	36	30	71.96	99.21
Outlier	113	20	58	52	51.40	98.73
CRISP	86	25	17	38	64.49	99.63
VarScan						
ComSeq	743	30	661	25	76.64	85.51
Poisson	60	18	11	58	45.79	99.76

Performance comparison of CAMBa to other methods using default parameters, on the Wheat data set after artificially increasing the coverage variance. The column abbreviations are as in Table 1. All methods are evaluated on their ability to predict the carrier of each candidate mutation, except CRISP, which cannot make such predictions. We were unable to report results for VarScan due to technical issues in generating a modified pileup of reads.

In the second study, we gradually increase the coverage level variance across libraries in the wheat experiment by selectively discarding base reads. We set the new coverage level of each library on a given gene to be the coverage ratio of that library to the library with the highest coverage on that gene, raised to the scaling factor *s *= 5, multiplied by the coverage of the highest coverage library for that gene. To reach the desired coverage level for a given gene on a given library, we discard each base call with fixed probability.

This level of coverage variance is comparable to that of HLP1 in Figure 3. We propose that the likeliest reason for the advantage we see in CAMBa's performance over the Outlier method, as shown in Table 4 is due to its insensitivity to coverage variability in the data, an effect of both its explicit use of coverage in the model, and the greater signal sensitivity imparted by the overlapping pool design.

**Table 4:** Performance comparison of CAMBa to other methods using default parameters on Rice with lowered variance

Method	Rice with lowered variance					
	Pred	Conf	FP	FN	Sens	Spec
CAMBa	73	10	9	6	91.43	99.95
Outlier	73	10	9	6	91.43	99.95
CRISP	0	0	0	70	0.00	100.00
VarScan	3	0	3	70	0.00	99.98
ComSeq	8106	10	8042	6	91.43	51.25
Poisson	6247	8	6196	19	72.86	62.44

Performance comparison of CAMBa to other methods using default parameters, on the Rice data set, after excluding HLP1 to lower the mean coverage variance. The column abbreviations are as in Table 1. All methods are evaluated on their ability to predict the carrier of each candidate mutation, except CRISP, which cannot make such predictions.

We note that while the rice experiment uses the mutagen MNU and the wheat experiment uses EMS, the choice of mutagen does not seem to have a significant effect on either the overall mutagenesis rate or the proportion of canonical versus non-canonical mutations [2].

### Attributing Performance Advantage to Model Features

Coverage variability does not fully explain CAMBa's performance, as its advantage is evident on both the Rice and Wheat data (although CAMBa's advantage is always smaller on Wheat). The poor quality of the Rice sequencing data is likely the reason that VarScan and CRISP return so few predictions on that data set. Other methods did not perform as well as CAMBa on either data set. Here we discuss a number of strategies and assumptions whose presence or absence in the various competing methods could account for the observed performance advantage of CAMBa.

CAMBa's advantage compared to competing methods is attributable to the combination of its comprehensive modeling of the data variability over all libraries, effective use of the pooled design, and its ability to simultaneously consider all candidate configurations with and without mutations in order to determine the best one. While each of the comparison methods have some of these properties, none has all three together.

Not explicitly considering coverage levels and instead relying directly on base change frequencies (e.g. ComSeq and Outlier), results in decreased performance relative to CAMBa on data sets with increased coverage variance. By contrast, the Poisson method, VarScan, CRISP, and CAMBa all consider coverage directly. CAMBa, Outlier, and Poisson are the only methods that were designed specifically for a multi-dimensional gridded pooling scheme. VarScan is run on individual libraries, CRISP is designed to run on one dimension, and ComSeq was designed with the flexibility that it may be run on a computationally optimal pooling scheme. Only CAMBa and ComSeq simultaneously consider a set of models of different possible mutant configurations and the model of the configuration in which there is no mutation and then use base change information across all libraries to choose the best model. In contrast, the Poisson and Outlier methods simply test for rejection of the null hypothesis of a configuration with no mutation. Interestingly, CRISP takes into consideration the model of a configuration with no mutation and models of mutant configurations, but it individually tests for rejection of the null hypothesis, rather than using an integrated approach to determine the best configuration.

## Conclusions

We demonstrated that our probabilistic method, which explicitly takes into account the bi-dimensional, overlapping pools experimental setup, and sequence coverage at each position for each library, can effectively discover rare mutations in large populations, as well as the individuals that carry them. It also has a performance advantage over other methods for detecting mutations from high-throughput sequencing of a TILLING population when there is significant coverage variability over libraries or lower quality data. More generally, it follows from our experiments that accounting for sequencing coverage variability can improve mutation detection in overlapping DNA pools. It would be interesting to work out the relationship between coverage depth and pool size. Likewise, we demonstrated that an overlapping pooling scheme, beyond offering carrier identification, also yields increased sensitivity of mutation detection when the data is less than ideal. This work implies a possible association between the amount of pool overlap (i.e. pool design code efficiency, or dimensionality of an experimental setup) and detection sensitivity, which deserves closer attention, especially for experiments on larger populations.

There are several directions in which our tool can be improved. We can add to our model an explicit account for position dependence of the mutations. Also, we can extend the model to allow multiple mutations at any given position (because of prior estimates of such events, we suspect that those improvements together will yield less than 10% increase in efficacy). We plan to continue using and improving CAMBa in our TILLING-by-Sequencing experiments. As other technological issues like higher throughput and sequence tagging get introduced into our pipeline, the issues of coverage sufficiency and higher multi-dimensional TILLING will be addressed.

We note that properly accounting for coverage variability may improve results in other genomics problems benefiting from 2nd generation sequencing, like sequence mapping, genome assembly, and motif finding.

## Competing interests

The authors declare that they have no competing interests.

## Authors' contributions

The TILLING-by-Sequencing technology was developed by LC. VM, LC, and VF conceived the computational framework, VM and VF designed the studies. VM developed the software and ran the experiments. VM and VF wrote the paper, LC helped in editing. All authors read and approved the final manuscript.

## Endnotes

^1^This is supported by evidence from a previous TILLING experiment in tetraploid wheat using the mutagen EMS, where Slade et al. [3] identified 50 positions for which at least one of the 768 individuals contained a mutation but only 3 for which there was a mutation in two individuals. In rice, which has a significantly lower expected mutagenesis rate at each possible reference base [2-4], we expect an even smaller percentage of the positions for which there is a mutation in one individual to have a mutation in more than one individual.

^2^These sets of confirmed mutations come from predictions using prior iterations of our approach, and prior experimental approaches, using CEL I, on these data sets, all of which were subsequently confirmed with PCR amplification.
